# Characterisation and localisation of the opsin protein repertoire in the brain and retinas of a spider and an onychophoran

**DOI:** 10.1186/1471-2148-13-186

**Published:** 2013-09-08

**Authors:** Bo Joakim Eriksson, David Fredman, Gerhard Steiner, Axel Schmid

**Affiliations:** 1 Department of Neurobiology, Faculty of Life Sciences, University of Vienna, Vienna, Austria; 2 Department of Molecular Evolution and Development, Faculty of Life Sciences, University of Vienna, Vienna, Austria; 3 Department of Integrative Zoology, Faculty of Life Sciences, University of Vienna, Vienna, Austria

**Keywords:** Opsins, Onychophora, Spider, Expression pattern, Ciliary opsin, Transcriptome

## Abstract

**Background:**

Opsins have been found in the majority of animals and their most apparent functions are related to vision and light-guided behaviour. As an increasing number of sequences have become available it has become clear that many opsin-like transcripts are expressed in tissues other than the eyes. Opsins can be divided into three main groups: rhabdomeric opsins (r-opsins), ciliary opsins (c-opsins) and group 4 opsins. In arthropods, the main focus has been on the r-opsins involved in vision. However, with increased sequencing it is becoming clear that arthropods also possess opsins of the c-type, group 4 opsins and the newly discovered arthropsins but the functions of these opsins are unknown in arthropods and data on their localisation is limited or absent.

**Results:**

We identified opsins from the spider *Cupiennius salei* and the onychophoran *Euperipatoides kanangrensis* and characterised the phylogeny and localisation of these transcripts. We recovered all known visual opsins in *C. salei,* and in addition found a peropsin, a c-opsin and an opsin resembling *Daphnia pulex* arthropsin. The peropsin was expressed in all eye types except the anterior median eyes. The arthropsin and the c-opsin were expressed in the central nervous system but not the eyes. In *E. kanangrensis* we found: a c-opsin; an opsin resembling *D. pulex* arthropsins; and an r-opsin with high sequence similarity to previously published onychophoran onychopsins. The *E. kanangrensis* c-opsin and onychopsin were expressed in both the eyes and the brain but the arthropsin only in the brain.

**Conclusion:**

Our novel finding that opsins of both the ciliary and rhabdomeric type are present in the onychophoran and a spider suggests that these two types of opsins were present in the last common ancestor of the Onychophora and Euarthropoda. The expression of the c-opsin in the eye of an onychophoran indicates that c-opsins may originally have been involved in vision in the arthropod clade. The lack of c-opsin expression in the spider retina suggests that the role for c-opsin in vision was lost in the euarthropods. Our discovery of arthropsin in onychophorans and spiders dates the emergence of arthropsin to the common ancestor of Onychophora and Euarthropoda and their expression in the brain suggests a non-visual function.

## Background

Since the first opsin sequence was described thirty years ago [1,2] more than 2000 opsin sequences have been published [3]. Opsins of different types are expressed in multiple tissues, indicating that opsin-dependent photosensitivity is not restricted to the eyes or the central nervous system (CNS) [3-5]. The traditional view has been that, unlike in vertebrates where ciliary opsins (c-opsins) are responsible for vision, rhabdomeric opsins (r-opsins) are the main opsins responsible for vision in protostomes (we will hereafter refer to opsins responsible for vision, determined either by functional studies or inferred from their expression in photoreceptive cells of the eyes, as visual opsins). However, an increasing number of c-opsins are now being detected in protostomes; c-opsins are expressed in the brain of annelids and honeybees [5-7], and it was recently shown that brachiopod larvae express a c-opsin homologue and have photoreceptive cells in the cerebral ciliary type eye spots [8]. Within the arthropods, molecular biology has mainly been focused on just a few model species such as *Drosophila melanogaster*, *Tribolium castaneum* and *Parhyale hawaiensis,* but there are plans to sequence thousands of arthropod genomes [9]. As additional genomes and transcriptomes are sequenced, and the eyes of additional animal groups are investigated, additional c-opsins are likely to be found. The opsin repertoire in arthropods appears to be highly divergent; the number of opsins detected varies from just three, e.g. in spiders [10,11] to 46 in the recently sequenced genome of *Daphnia pulex*[12]. However, this variability may be due to differences in sampling methods (i.e. screening with degenerate PCR versus whole genome sequencing). In order to study the evolution of arthropod opsins and infer the gene composition of the ancestral arthropod, it is necessary to compare arthropods to more basal groups such as onychophorans and tardigrades. The latest phylogenetic analyses place onychophorans as a sister group to euarthropods [13]. Onychophorans have a pair of small eyes situated at the base of the antenna [14,15]. Recently, the sequence of one r-opsin homologue, named onychopsin, as well as phototactic behaviour were described in onychophorans [16] but no c-opsin was found. The documented presence of c-opsins in some arthropods such as the honeybee and the water flea (where these c-opsins are referred to as pteropsins), as well as in the lophotrochozoan representatives of annelids and brachiopods [6,7,12], suggests a loss in the onychophoran lineage. However, in onychophorans, the presence of a rudimentary cilium in the photoreceptive cells has been described [15,17], indicating instead that a c-opsin may be present in the onychophoran eye as well. To our knowledge there has been no report of cilia in the photoreceptive cells of cerebral eyes in euarthropods. It has been proposed that phototransduction were originally based on c-opsins, and that the ciliary phototransduction pathway evolved before the split of cnidarian and bilaterian animals [18]. Vision or photodetection by r-opsins and their associated signalling pathway, evolved later in protostomes, taking over the visual phototransduction [18]. In chelicerates several visual opsins have been described but so far no c-opsin [10,19]. Tardigrades are regarded as a sister group to onychophorans and euarthropods [13]. A study of the ultra-structure of tardigrade photoreceptive cells revealed both rhabdomeric and ciliary structures [20], however no opsin sequences of tardigrades are known today.

Arthropsins are a new set of r-opsins discovered in the sequenced genome of *D. pulex*[12]*.* The function and localisation of arthropsin transcripts are unknown and there has been no report of their presence in other arthropods.

Given that onychophorans are regarded as a sister group to euarthropods and that there is some support for placing chelicerates as basal within euarthropods [21], we decided to search for additional opsin homologues in the Australian onychophoran species *Euperipatoides kanangrensis* (Peripatopsidae) and in the Central American wandering spider *Cupiennius salei* (Ctenidae)*,* in order to shed some light on the evolution of arthropod opsins. By analysing sequences from a mixed tissue transcriptome of *E. kanangrensis* and transcriptomes specific for retina, CNS and mixed embryonic stages of *C. salei,* we discovered multiple novel opsins in both species. A c-opsin homologue was found in both species. Using RT-PCR on RNA isolated from the eye and brain of *E. kanangrensis* and by analysing transcriptomes as well as RT-PCR of *C. salei*, we detected these transcripts in both the eye and brain of *E. kanangrensis* and in the brain but not in the eyes of *C. salei*. We identified an r-opsin that is a putative arthropsin and, in both the spider and the onychophoran, was expressed in the CNS but not the eyes. In addition, we found a peropsin homologue in the spider but not in the onychophoran transcriptome. Peropsin, which was recently placed within the clade of group 4 opsins [4], has been described in several vertebrates, *Amphioxus* and in the jumping spider *Hasarius adansoni*[11] and is thought to function as a photoisomerase in chordates [5,22,23]. Based on these findings, we suggest that the last common ancestor of onychophorans and arthropods had at least four types of opsins: one visual r-opsin; one c-opsin that might have had a function in the eye; one r-opsin that is not expressed in the eyes and is related to the arthropsins of *D. pulex*; and a peropsin that was lost in the onychophoran lineage.

## Results

### Orthology of the genes

**Figure 1:**
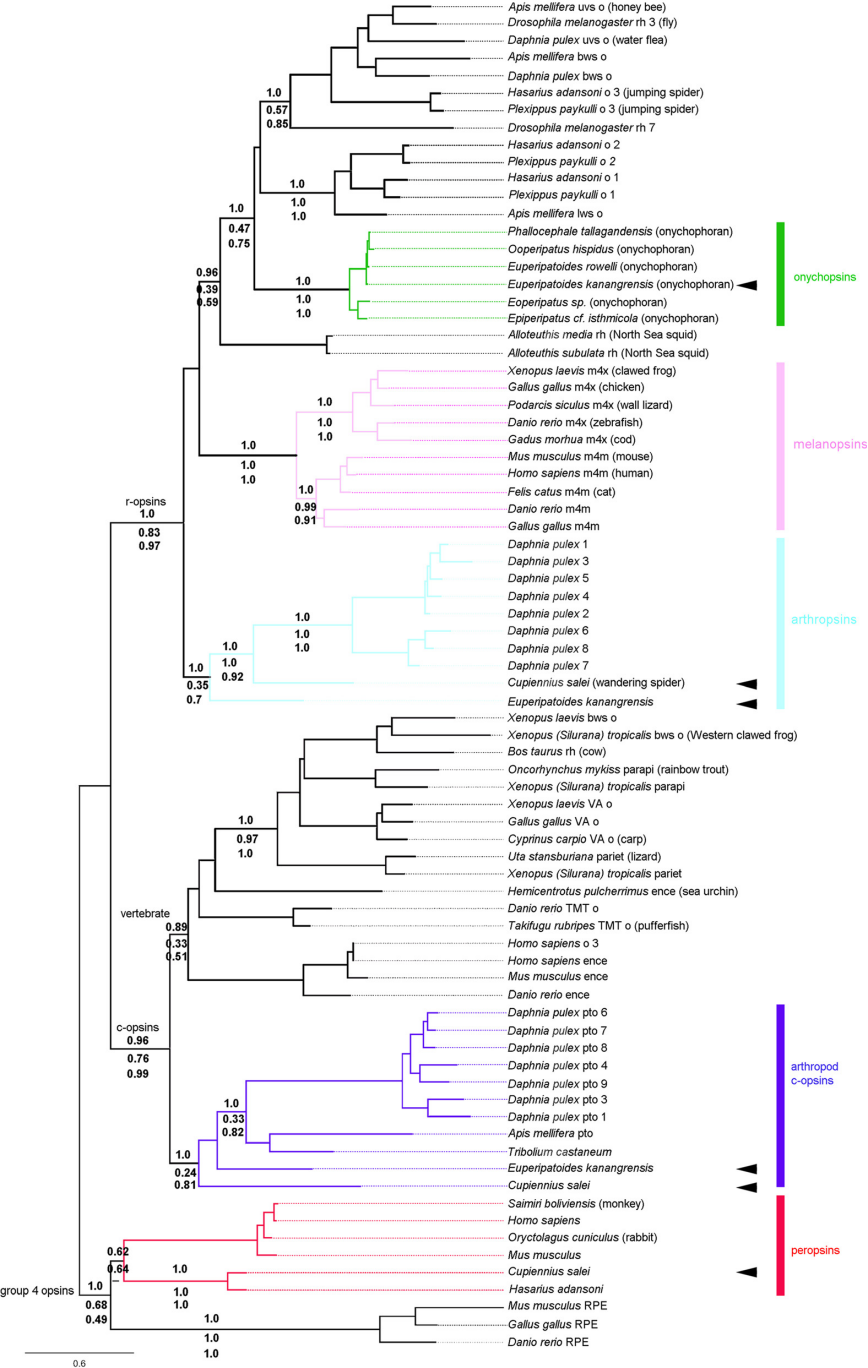
**Phylogenetic tree of opsins.** Phylogenetic reconstruction of c-opsins, group 4 opsins and r-opsins using Bayesian Inference as implemented in MrBayes 3.2.1 [24]. Every 100^th^ tree of 5,000,000 generations of 2x4 Markov chains under a WAG distribution model of amino acid substitutions was sampled. The Bayesian tree represents the half compatibility consensus of this sample after discarding 200,000 generations as burn-in. Opsin sequences were aligned with clustalW and regions outside of the 7 transmembrane domains were excluded. Numbers at nodes represent Bayesian posterior probabilities (top), parsimony bootstrap values (middle; 5,000 replicates in PAUP* 4.10b [2002]) and Maximum Likelihood (ML) bootstrap values (lower; 100 replicates in PHYML. The scale bar shows substitutions per site. The *C. salei* and *E. kanangrensis* positions are marked with arrowheads. The colours of the different groups are: onychopsins dark green, melanopsins pink, arthropsins light blue, arthropod c-opsins dark blue, peropsins red. The accession numbers of included species are provided in Additional file 5. Abbreviations, bws o = blue wavelength sensitive, ence = encephalopsin, gws o = green wavelength sensitive opsin, lws o = long wavelength sensitive opsin, m4m = melanopsin (opn4) mammalian-like, m4x = melanopsin (opn4) non-mammalian-like, o = opsin, parapi = parapinopsin, pariet = parietopsin, pto = pteropsin, rh = rhodopsin, RPE = RPE-retinal G protein-coupled receptor, TMT o = teleost multiple tissue opsin, uvs o = ultraviolet sensitive opsin, VA o = vertebrate ancient opsin.

**Figure 2:**
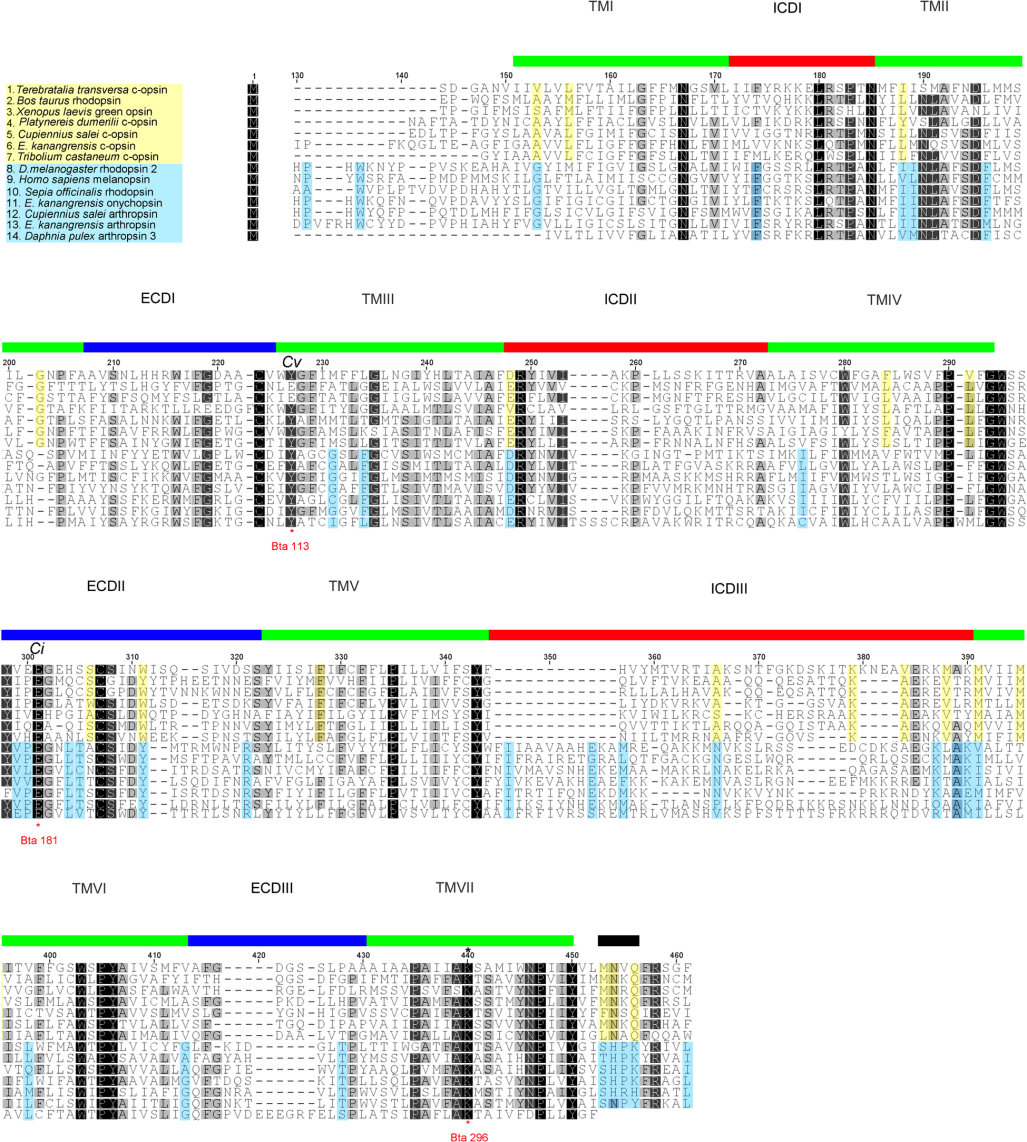
**Alignment of opsin sequences.** Alignment of a subset of c-opsins (yellow) and r-opsins (blue) shows sites with conserved amino acid residues specific for c-opsins (yellow shading) and r-opsins (blue shading). Green horizontal bars indicate transmembrane regions I to VII, blue horizontal bars indicate extracellular domains (ECDI-ECDIII), red horizontal bars indicate intracellular domains (ICDI-ICDIII) and a black horizontal bar indicates a region believed to interact with G-protein. Cv marks the site believed to harbour the counter ion (glutamic acid, E) residue in vertebrates [25] and Ci for invertebrates [26]. An asterisk marks the residue lysine (K) that binds the retinal. Bta 113, Bta 181, Bta 296 and asterisks indicate the bovine opsin numbering system. Different grey-scale shadings indicate the percentage of amino acids identical at the given position, black = 100%, dark grey = at least 90%, light grey = at least 75%.

We found three opsin-like genes in the transcriptome of the onychophoran species *E. kanangrensis* that we have designated as: Ek onychopsin, Ek c-opsin and Ek arthropsin. Ek onychopsin shows 98% identity on the nucleotide level (data not shown) to the recently published sequence of a closely related species *E. rowellii*[16], hence its designation as an onychopsin. Ek c-opsin groups with other c-opsins and forms, together with Cs c-opsin, a sister group to other arthropod c-opsins (pteropsins) in our phylogenetic analysis (Figure 1). Ek c-opsin also contains c-opsin-specific amino acid residues (Figure 2). The Ek arthropsin clearly groups with r-opsins, as a sister group to *Daphnia pulex* and *C. salei* arthropsins (Figure 1, Additional file 1) and contain r-opsin-specific amino acid residues (Figure 2).

We found six opsin-like genes in the *C. salei* transcriptomes of mixed retinas, mixed embryonic tissues and CNS. Three of these are known visual opsins (Cs-Rh1, -Rh2, -Rh3) and are described elsewhere [27]. We designated the remaining opsins as: Cs peropsin, Cs c-opsin and Cs arthropsin. Together with another spider (*H. adansoni*) peropsin, Cs peropsin forms a sister group to vertebrate peropsins (Figure 1). The Cs c-opsin groups with other c-opsins (see Ek c-opsin above). The Cs arthropsin groups with other r-opsins and forms a group with *D. pulex* arthropsins (Figure 1, Additional file 1). All the described opsin-like genes in *E. kanangrensis* and *C. salei* are 7 transmembrane proteins that contain a retinal binding lysine residue in transmembrane region VII and a putative counter ion glutamatic acid residue in the area between transmembrane region IV and V (Figure 2) [26]. In all presented opsins, the corresponding position of the presumed vertebrate counter ion residue in transmembrane region III is a tyrosine instead of a glutamatic acid residue like in vertebrates [25] (Figure 2). In an alignment of a subset of the sequences (Figure 2) used for the phylogenetic analysis, the c-opsins have conserved amino acid residues distinct from the r-opsins. Specifically conserved in the c-opsins are the amino acid residues directly downstream of transmembrane domain VII and near transmembrane domain VI. Those residues have been suggested as being important for G protein interaction [28,29].

### Orthologous sequences in other arthropods

In order to find orthologous sequences for Cs arthropsin and Cs peropsin we searched the published genomes of the chelicerates *Ixodes scapularis* and *Metaseiulus occidentalis*; the insects *Anopheles gambiae*, *Apis mellifera* and *Tribolium castaneum;* and the centipede *Strigamia maritima.* We could not find any sequences similar to Cs-arthropsin or *Daphnia pulex* arthropsins. We did not find any opsin homologue in the genome of the centipede *S. maritima*. We used the above listed genomes plus the genome of the crustacean *Daphnia pulex* to search for sequences similar to Cs-peropsin. We found two short fragments in the tick *I. scapularis* and two sequences in the tick *M. occidentalis* that include the start methionine as well as all 7 transmembrane regions, but no matching sequences were found in the other euarthropod species. The two short fragments, 96 and 97 amino acids in length, found in the tick *I. scapularis* could indicate incomplete annotation or that they are pseudogenes. The two peropsin-like sequences in the other tick *M. occidentalis* show ca. 30% amino acid sequence identity to Cs-peropsin. The sequences of *M. occidentalis* were, according to the NCBI database, automatically annotated as peropsin-like sequences (NCBI Reference Sequence: XP_003744578.1 and XP_003744590.1).

### Localisation of spider opsin transcripts

*C. salei* peropsin was detected in all of the transcriptomes (retinas, CNS and embryonic). Since the retina transcriptome was made from pooled retinas of all eye types we performed reverse transcription PCR (RT-PCR) using RNA from dissected retinas of each eye type as well as RNA isolated from CNS. *C. salei* peropsin transcripts were detected in the CNS as well as in the secondary eyes (PM, AL, PL eyes), but not in the primary eyes (AM eyes) (Additional file 2). *C. salei* c-opsin and arthropsin were detected in the transcriptomes of the CNS only.

### Localisation of onychophoran opsin and onychophoran retinal rod sensitive cyclic GMP phosphodiesterase transcripts

The transcriptome of *E. kanangrensis* was made from mixed tissue. In order to localise the transcripts of onychophoran opsins we performed RT-PCR using RNA from dissected eyes and brains. *E. kanangrensis* onychopsin and c-opsin were detected in both eye and brain samples but *E. kanangrensis* arthropsin only in brains (Additional file 3A-D, G). In order to assess the possibility of a ciliary phototransduction chain in the onychophoran eye we screened for cGMP PDE in the *E. kanangrensis* transcriptome and found a sequence that matched other retinal rod sensitive cyclic GMP phosphodiesterases in the NCBI database (closest match *Apis mellifera* cGMP PDE). The RT-PCR reaction for *E. kanangrensis*-cGMP PDE using either eye or brain cDNA as a template gave a product in both cases (Additional file 3E-F).

## Discussion

### Visual opsin evolution

The onychophoran photoreceptor cells contain microvillar membrane extensions and only a rudimentary cilia and are therefore categorised as rhabdomeric [15,17]. The rhabdomeric photoreceptor cells and the expression of an r-opsin (onychopsin) in the eye suggest that the *E. kanangrensis* onychopsin is the opsin responsible for vision in the onychophorans as previously suggested [16]. Hence, we use the term visual opsin in order to distinguish it from opsins with non-visual functions as concluded by expression in tissues other than the eye. It is likely that the *E. kanangrensis* c-opsin has a light-driven function other than vision in the onychophoran eye, perhaps a modulating function. However, great caution should be taken in making speculations regarding the opsin responsible for image formation in *E. kanangrensis* since we lack detailed expression data at the cellular level. Our results are compatible with the view put forward by Hering and co-workers (2012), that one ancestral visual opsin in arthropods was duplicated in euarthropods. It is also likely that losses have occurred; in a search for opsins in the available genome of the eye-less centipede *Strigamia maritima* we failed to find any opsin homologue.

### Ciliary opsins

It has been suggested that the photoreceptors evolved from dermal cells that contained both a cilium and microvilli (the mixed type) [30]. Photoreceptors of both the ciliary and microvillar type have been found in annelids [31,32], molluscs [33,34] and tardigrades [20]. The onychophoran photoreceptor cells have microvillar membrane enlargements but the cells also contain rudimentary cilia [15,17]. There are also reports of ciliary photoreceptors in the organ of Bellonci, an organ attached to the eye stalk and suggested to have photoreceptive functions, of the crustacean shrimp *Paratya rasmaniensis*[35]. The cerebral photorecepors of the brachiopod *Terebratella transversa* are of the ciliary type and the associated opsin was classified as a c-opsin by phylogenetic analysis [8]. However, to our knowledge, cerebral eyes with ciliary components have not been observed in euarthropods. A recent ultrastructural investigation of a pycnogonid, a basal euchelicerate or euarthropod, reported rhabdomeric structures in the photoreceptors [36]. Our investigation is the first report of the expression of an opsin of the ciliary type in the eyes of an onychophoran. Cilia are present in the embryonic ectoderm and in the hypocerebral organ of *E. kanangrensis*[37,38]. However, these cilia disappear in the adult, and only a few rudimentary cilia can be detected in the adult hypocerebral organ. The hypocerebral organ of onychophorans is a paired structure ventral to the brain. Its function is unknown, but a glandular role has been suggested [38,39]. The hypocerebral organ invaginates from the neuroectoderm and contains microvillar extensions in addition to cilia. The presence of ciliary structures in the rhabdomeric eyes and ciliary as well as rhabdomeric opsins in the eye of the onychophoran *E. kanangrensis* supports the suggestion by Salvini-Plawen (2008) that photoreceptors evolved from dermal photosensitive cells that possessed both types of opsins and cellular enlargement structures. Currently, the c-opsins of Euarthropoda have only been described for the bee *Apis mellifera*[7], the water flea *Daphnia pulex*[12], the mosquito *Aedes aegypti*[40] and the monarch butterfly *Danaus plexippus*[41]. In addition, a c-opsin sequence for the beetle *Tribolium castaneum* has been deposited in NCBI databases but it is likely that, as more arthropod genomes are annotated, additional c-opsin sequences will be described. In the honey bee the ciliary type of opsin is termed pteropsin and is expressed in a small number of cells in the protocerebrum [7]. There is no data on the expression pattern in *D. pulex* and *A. aegypti.* In *C. salei* we could only detect the c-opsin in the transcriptome of the adult CNS. The absence of c-opsin expression in the transcriptome of the mixed embryonic stages implies that its function is restricted to hatched spiders. There is no report of any ciliary structure in the eyes of *C. salei*[42]. It thus appears that both ciliary structures and c-opsin expression were lost in the spider eyes, while onychophorans retain a rudimentary cilium in their photoreceptors and express c-opsin in the eye. Tardigrades appear to have photoreceptors of the mixed type [20] although the function of its ciliary extensions in light detection are doubted by the author of that investigation. However, in our view, the ancestral gene complement underlying arthropod photosensitivity was a photoreceptor of the mixed type that has gradually been lost in the lineage toward Euarthropoda and Onychophora, and completely lost in euarthropods, given that the most recent view of arthropod phylogeny [13] holds true.

### Brain specific r-opsins or arthropsins

The non-visual spider and onychophoran r-opsins found in this study group with *D. pulex* arthropsins. The *D. pulex* arthropsins were recently described in the publication of the *D. pulex* genome [12]. Their expression patterns and functions are unknown [12]. We searched the published genomes of other arthropods, but could not find any similar opsin sequences. However, with many more arthropod genomes being sequenced, it is possible that arthropsins will be discovered in additional arthropod taxa in the future. The arthropsins of both spider and onychophoran were expressed in CNS tissue but not in the eyes, suggesting that they are not involved in vision but likely some other light driven effect, since they both contain a lysine at the retinal binding site in transmembrane region 7.

### Peropsins

Peropsins are members of the group 4 opsins. They were first described in the human retina [23] and experiments on a peropsin homologue in amphioxus indicate that it might be a photoisomerase [22]. In arthropods, peropsin has so far only been described in a spider [11], however, the very weak branch support values between spider and vertebrate peropsins in our investigation indicates that spider peropsins are very distant from vertebrate sequences (Figure 1). Peropsin expression was absent in the *C. salei* AM eyes and we did not find any peropsin homologue in the *E. kanangrensis* transcriptome. In the jumping spider *H. adansoni,* peropsin was detected by *in situ* hybridisation in the AM eye (the secondary eyes were not included in the investigation) but in an area outside of the photoreceptor region [11]. The expression of peropsins outside of the photoreceptor region in *H. adansoni* indicates that the peropsin does not function as a photoisomerase. We await the more precise localisation of Cs peropsin in the secondary eyes of *C. salei* by in situ hybridisation before discussing its potential role in the vision of the secondary eyes. Nevertheless, differences in morphology and function between the primary eyes and the secondary eyes of *C. salei* have been characterized [43], and hence, differences on the molecular level are to be expected. Despite repeated RT-PCR on *C. salei* retina cDNA we were unable to amplify any peropsin product from the AM eyes.

## Conclusions

**Figure 3:**
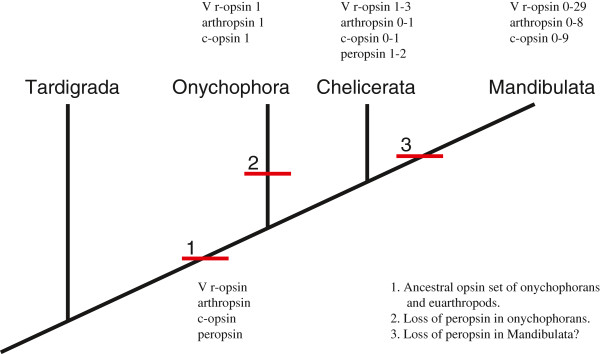
**The original opsin set.** Tree of arthropods showing the ground-pattern of the opsin set for Onychophora and Euarthropoda: arthropsin, visual r-opsin (V r-opsin), c-opsin and peropsin and what is presently known of the opsin set in each of the groups Onychophora, Chelicerata and Mandibulata. We would like to stress that the suggested loss of peropsin in the Mandibulata is highly uncertain at this point, due to limited sequence availability. Further data on the genomes of additional mandibulates may certainly reveal a peropsin homologue. Sources for opsin numbers are as follows: Onychophora, this investigation and [16]; Chelicerata, NCBI database search in this investigation and [10,11]; Mandibulata, NCBI database search in this investigation and [12].

In this investigation we have found three opsins in *E. kanangrensis*: one presumed visual opsin expressed in both the eye and brain; one c-opsin expressed in both the eye and brain; and one r-opsin expressed in brain but not in the eye. An earlier investigation of five onychophoran species only recovered one onychopsin sequence from each species [16]. One explanation for not finding any of the other opsins is that they have been lost in those species, another is that the transcripts are rare and the transcriptome sequencing depth insufficient to recover them. Additionally, different methods of transcriptome sequencing and assembly may produce different results. In *C. salei* we found, in addition to three visual opsins discussed elsewhere, three presumably non-visual opsins: one peropsin expressed in the secondary eyes and in the brain; one c-opsin expressed in the brain but not in the eyes; and an r-opsin, designated as arthropsin, expressed in the brain but not in the eyes. The *C. salei* and *E. kanangrensis* c-opsins are homologous to c-opsins of other arthropods like the multiple pteropsins in *D. pulex* and the pteropsin described in *A. mellifera*. The *C. salei* peropsin has homologues in vertebrates as well as in some euarthropods and it is therefore likely that this gene has been lost in the lineage to onychophorans. When additional arthropod genomes and transcriptomes are sequenced it is possible that peropsins as well as arthropsins will be described in the different euarthropod groups, e.g. crustaceans and pycnogonids for which the genome sequence representation is either limited (crustaceans) or completely lacking (pycnogonids). However, in the recently sequenced genome of the eye-less centipede *Strigamia maritima* we did not find any opsin sequence at all, neither visual nor non-visual, indicating that in *S. maritima* the need for light induced stimuli involving opsins has been lost. Incidentally, we also did not find any sequences homologous to cryptochrome; cryptochrome is a light sensitive protein involved in circadian rhythm in several species as well as involved in light responses in plants [44]. We postulate that the original set of arthropod opsins was composed of at least four opsin-like genes: one visual opsin; one peropsin; one arthropsin; and one c-opsin. This opsin set then underwent reduction in some lineages, with an extreme example being the complete loss of opsins in the Centipede *Strigamia maritima*, and expansion in other lineages, such as that resulting in 46 opsins in the crustacean *D. pulex* (Figure 3).

## Methods

### Animal husbandry

Adult *C. salei* raised in our laboratory in Vienna were used for the extraction of RNA. The spiders were kept individually in glass jars (25 cm high, 14.5 cm in diameter) and fed once per week on flies. The temperature was kept at 22°C and the relative humidity above 60%. After mating, females produced a cocoon filled with hundreds of eggs. In order to get RNA from most stages of development, the cocoons were removed, opened and a number of embryos removed each day for total RNA extraction. RNA from each day, from 3 to 30 days after egg laying, was obtained and subsequently used for either sequencing or cDNA synthesis. Adult spiders were dissected to obtain retinas and CNS for RNA extraction.

Female *Euperipatoides kanangrensis* Reid, 1996 were collected in Kanangra Boyd National Park, NSW, Australia 33° 59′S 150° 08′E. Females were kept in containers with dampened sphagnum moss at 13°C and were fed first instar locusts or crickets once every second week. RNA was extracted from mixed embryonic stages, CNS and eyes. The NSW Government Department of Environment and Climate Change provided a permit SL100159 to collect onychophorans at Kanangra Boyd National Park.

### Screening for genes

Total RNA was isolated with the Trizol method (Invitrogen/Life technologies) from mixed embryonic tissue, CNS from adults and adult retinas. The extracted RNA was sent to Genecore (EMBL, Heidelberg) for sequencing (Illumina hi-seq, paired-end 100 bp, not normalised). Following quality filtering of reads and *de novo* transcript assembly using Oases v0.2.08 (**ref** PMID: 22368243), we searched the resulting transcript database for matches to a diverse set of opsin proteins downloaded from NCBI. BLAST searches and sequence analysis were done with the computer program Geneious versions 5.6.6-6.1.6 created by Biomatters (http://www.geneious.com/). Opsin sequence orthology was established by aligning the identified *C. salei* and *E. kanangrensis* sequences to invertebrate and vertebrate r-opsins, c-opsins and group 4 opsins We also screened the *E. kanangrensis* transcriptome for retinal rod rhodopsin sensitive cGMP 3′,5′-cyclic phosphodiesterase subunit delta (Ek-cGMP PDE), which is part of the ciliary phototransduction pathway. Opsin sequences were in all cases aligned with clustalW and regions outside of the 7 transmembrane domains as well as gaps were excluded. A Bayesian tree was constructed using MrBayes with a WAG distribution model of amino acid substitutions [24]. We also determined branch support by maximum likelihood bootstrap with the PhyML software [45-49] and a parsimony bootstrap in PAUP* [50] (Version 4. Sinauer Associates, Sunderland, Massachusetts). The alignments are provided in the Additional file 4 and the accession numbers of the included sequences in Additional file 5.

### Reverse transcription PCR

Total RNA from four *C. salei* individuals was isolated from dissected retinas from each of the four eye types: anterior median (AM); posterior median (PM); anterior lateral (AL); and posterior lateral (PL), and from the anterior CNS. Total RNA from *E. kanangrensis* was isolated from the dissected eyes and brains of 7 adult animals. The eyes of onychophorans are situated on a protrusion away from the brain [15,51,52]. Therefore, eyes free from contaminating brain tissue could be isolated relatively easily using a pair of microsurgical scissors. The brains were dissected from the body and any muscle tissue, integument or nervous tissue posterior to the circumoesophageal connectives and the distal parts of the antennae were removed. RNA was extracted with the Trizol method (Invitrogen/Life technologies) and used for reverse transcription using Thermoscript (Invitrogen/Life technologies). The resulting cDNA was used as template in subsequent polymerase chain reactions (PCR). Primers were constructed based on opsin sequences found in the *C. salei* and *E. kanangrensis* transcriptome database using the software primer3 [53]. The following primers were used: Cs peropsin forward 5′ CGGTTTG TTCCCTGTGATTC 3′ Cs peropsin reverse 5′ AGGCCATGGTGGATAAAATG 3′; Cs arthropsin forward 5′ TTCTTGACGGGGAACTCATC 3′, Cs arthropsin reverse 5′ ACTG C CACGCCGAGATATAC ′3; Cs c-opsin forward 5′ AGAGGCATCCAACTCAACGTCCA 3′; Cs c-opsin reverse 5′ TCTCGGTAGTGGCACTTTTATCTCCA 3′; Ek onychopsin forward 5′ ACCACTCAGCAGATGACCAGACT 3′; Ek onychopsin reverse 5′ TTGA T G CCTGAACTGTGGGTGGT 3′; Ek onychopsin nested forward 5′ TGTCGGAA CCC A GT GCAGCAG 3′; Ek onychopsin nested reverse 5′ TGCCAGTGAGGACTGCC TTGA3′; Ek c-opsin forward 5′ GCCGTAGCGCTGCTGGACTT 3′; Ek c-opsin reverse 5′ ACTACAACC ATGGGTGAACTTGTGTC 3′; Ek c-opsin nested forward 5′ TCTCCAAAC CATTTG CTG CTGCC 3′; Ek c-opsin nested reverse 5′ GGGCCAGCAAGTGGTTCCCT 3′; Ek arthropsin forward 5′ CGTGTCGCGTATCAAAGTTA 3′; Ek arthropsin reverse 5′ ACC CAACTCA ACATCAGCAGTGGA 3′; Ek arthropsin nested forward 5′ ACTATCCACCAG TCAGT AAGGAGGC 3′; Ek arthropsin nested reverse 5′ TGTTCCTGAAGGTTTC TTA ACCA 3′; Ek cGMP PDE forward 5′ ACGTGTGTTTGTCAACTCCG; Ek cGMP PDE reverse 3′ TCCATTGGTCTGCTTATCGACA. A PCR reaction was performed with primers for Cs peropsin on cDNA from each of the four eye types as well as CNS using GoTaq Flexi DNA Polymerase (Promega). The opsin sequences of *C. salei* have been submitted to EMBL GeneBank and have the following accession numbers: Cs peropsin, [EMBL: HF566406]; Cs c-opsin, [EMBL:HF566407]; Cs arthropsin, [EMBL:HF566408]. PCR and nested PCR reactions were performed on cDNA from eyes and brains of *E. kanangrensis* using GoTaq Flexi DNA Polymerase (Promega). The opsin sequences of *E. kanangrensis* have been submitted to EMBL GeneBank and have the following accession numbers: Ek onychopsin, [EMBL: HF566403]; Ek c-opsin, [EMBL: HF566404]; Ek-arthropsin, [EMBL: HF566405]. The Ek-cGMP PDE has the accession number: [EMBL: HF583289]. Submitted sequences of all spider and onychophoran opsin genes were confirmed by sequencing of cloned RT-PCR products.

### Ethics

The experiments in this investigation did not require any approval by an ethical committee.

## Availability of supporting data

Supporting data are included as additional files.

## Competing interests

The authors declare that they have no competing interests.

## Authors’ contributions

BJE conceived and designed the study, carried out the molecular genetic studies, participated in the sequence alignment and phylogenetic analyses and wrote the manuscript. DF assembled the transcriptomes and participated in writing the manuscript. GS participated in aligning the sequences and in the phylogenetic analyses. AS participated in writing the manuscript. All authors read and approved the final manuscript.

## Supplementary Material

Additional file 1: Figure S1 Phylogenetic reconstruction of c r-opsins. The three is from Bayesian likelihood analysis using MrBayes with a WAG distribution model of amino acid substitutions, half compatibility consensus from 1,100,000 replicates. The numbers above the branches are posterior probabilities from Bayesian analysis and the number below branches are bootstrap support from a maximum likelihood analysis with PhyML with 100 replicates. Opsin sequences were aligned with clustalW and regions outside of the 7 transmembrane domains were excluded. Bta rh (*Bos taurus*) rhodopsin was used as an outgroup. *C. salei* and *E. kanangrensis* protein names are coloured blue and brown respectively. Scale bar show substitutions per site. Species included in the analysis are a subset of the species used in Figure 1. See Figure 1 for abbreviations. Click here for file

Additional file 2: Figure S2 Image of a gel electrophoresis run with rtPCR products from Cs peropsin on templates from the different retina types and CNS. There was no peropsin detected in the AM eyes. The faint signal in the lane of AL reflect the small size of this eye. Abbreviations, AM = anterior median eyes, AL = anterior median eyes, -C = negative control, Kb = kilo base pairs, PL = posterior lateral eyes, PM = posterior median eyes. Click here for file

Additional file 3: Figure S3 Image of a gel electrophoresis run with rt PCR products from Eka c-opsin, eka cGMP PDE (retinal rod rhodopsin-sensitive cGMP 3’,5’-cyclic phosphodiesterase subunit delta), Eka arthropsin and Eka onychopsin an template from eye and brain. Click here for file

Additional file 4 **ClustalW alignement of opsin sequences used for doing the phylogenetic analyses that is represented in the trees in Figures **1** and Additional file **1**.** Click here for file

Additional file 5 **Table with species names opsin designation and accession number of the sequences included in the phylogenetic analyses that is represented in the trees in Figures **1** and Additional file **1**.** Click here for file
