# Supplementary material for: Characterisation and localisation of the opsin protein repertoire in the brain and retinas of a spider and an onychophoran
Source: BMC Evol Biol. 2013 Sep 8;13:186. doi: 10.1186/1471-2148-13-186 (PMC3851285; doi:10.1186/1471-2148-13-186)
Supplement: Additional file 1: Figure S1 — Phylogenetic reconstruction of c r-opsins. The three is from Bayesian likelihood analysis using MrBayes with a WAG distribution model of amino acid substitutions, half compatibility consensus from 1,100,000 replicates. The numbers above the branches are posterior probabilities from Bayesian analysis and the number below branches are bootstrap support from a maximum likelihood analysis with PhyML with 100 replicates. Opsin sequences were aligned with clustalW and regions outside of the 7 transmembrane domains were excluded. Bta rh (Bos taurus) rhodopsin was used as an outgroup. C. salei and E. kanangrensis protein names are coloured blue and brown respectively. Scale bar show substitutions per site. Species included in the analysis are a subset of the species used in Figure 1. See Figure 1 for abbreviations. [file 1471-2148-13-186-S1.pdf]

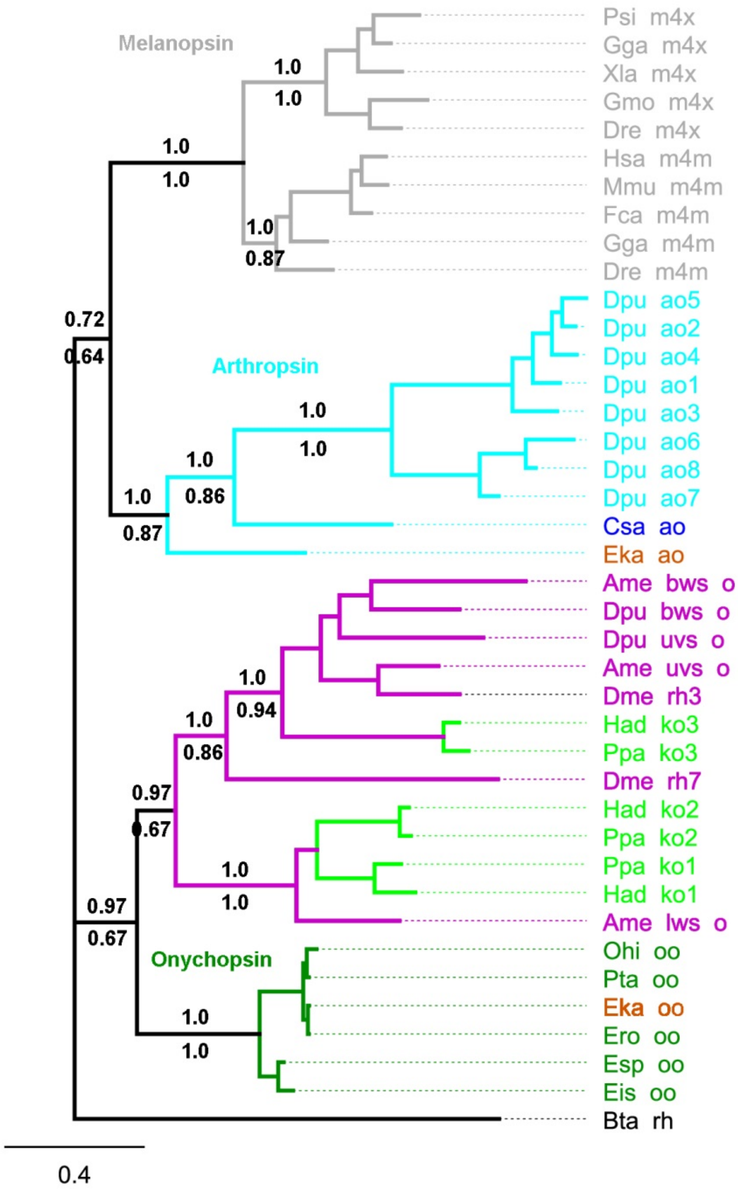

Additional figure S1 Phylogenetic reconstruction of c r-opsins. The three is from Bayesian likelihood analysis using MrBayes with a WAG distribution model of amino acid substitutions, half compatibility consensus from 1,100,000 replicates. The numbers above the branches are posterior probabilities from Bayesian analysis and the number below branches are bootstrap support from a maximum likelihood analysis with PhyML with 100 replicates. Opsin sequences were aligned with clustalW and regions outside of the 7 transmembrane domains were excluded. Bta rh (*Bos taurus*) rhodopsin was used as an outgroup. *C. salei* and *E. kanangrensis* protein names are coloured blue and brown respectively. Scale bar show substitutions per site. Species included in the analysis are a subset of the species used in figure 1. See figure 1 for abbreviations.
