# Supplementary material for: Characterisation and localisation of the opsin protein repertoire in the brain and retinas of a spider and an onychophoran
Source: BMC Evol Biol. 2013 Sep 8;13:186. doi: 10.1186/1471-2148-13-186 (PMC3851285; doi:10.1186/1471-2148-13-186)
Supplement: Additional file 2: Figure S2 — Image of a gel electrophoresis run with rtPCR products from Cs peropsin on templates from the different retina types and CNS. There was no peropsin detected in the AM eyes. The faint signal in the lane of AL reflect the small size of this eye. Abbreviations, AM = anterior median eyes, AL = anterior median eyes, -C = negative control, Kb = kilo base pairs, PL = posterior lateral eyes, PM = posterior median eyes. [file 1471-2148-13-186-S2.pdf]

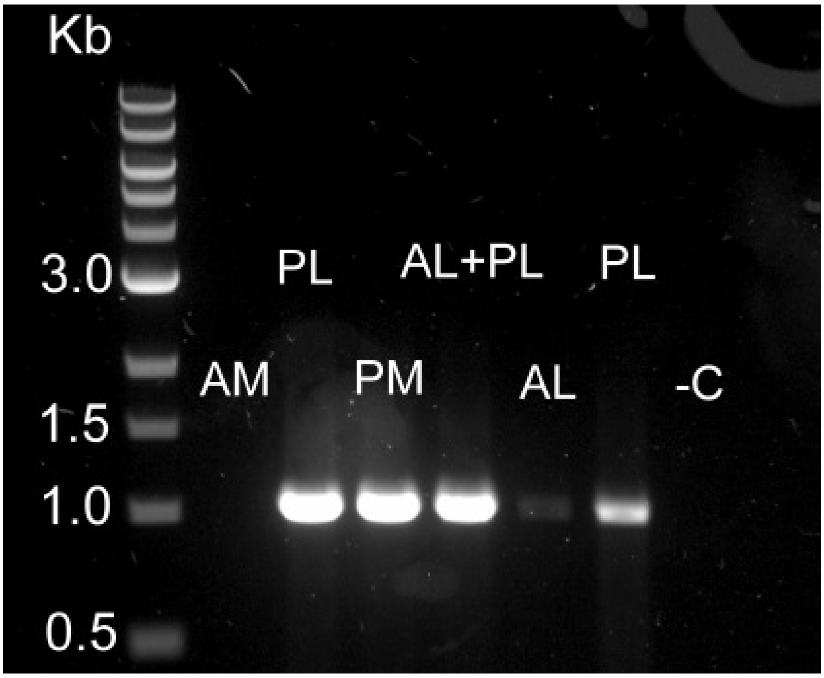

Additional figure S2. Image of a gel electrophoresis run with rtPCR products from Cs peropsin on templates from the different retina types and CNS. There was no peropsin detected in the AM eyes. The fain signal in the lane of AL reflect the small size of this eye. Abbreviations, AM = anterior median eyes, AL = anterior median eyes, -C = negative control, Kb = kilo base pairs, PL = posterior lateral eyes, PM = posterior median eyes
