# Supplementary figures and images for: Characterisation and localisation of the opsin protein repertoire in the brain and retinas of a spider and an onychophoran
Source: BMC Evol Biol. 2013 Sep 8;13:186. doi: 10.1186/1471-2148-13-186 (PMC3851285; doi:10.1186/1471-2148-13-186)

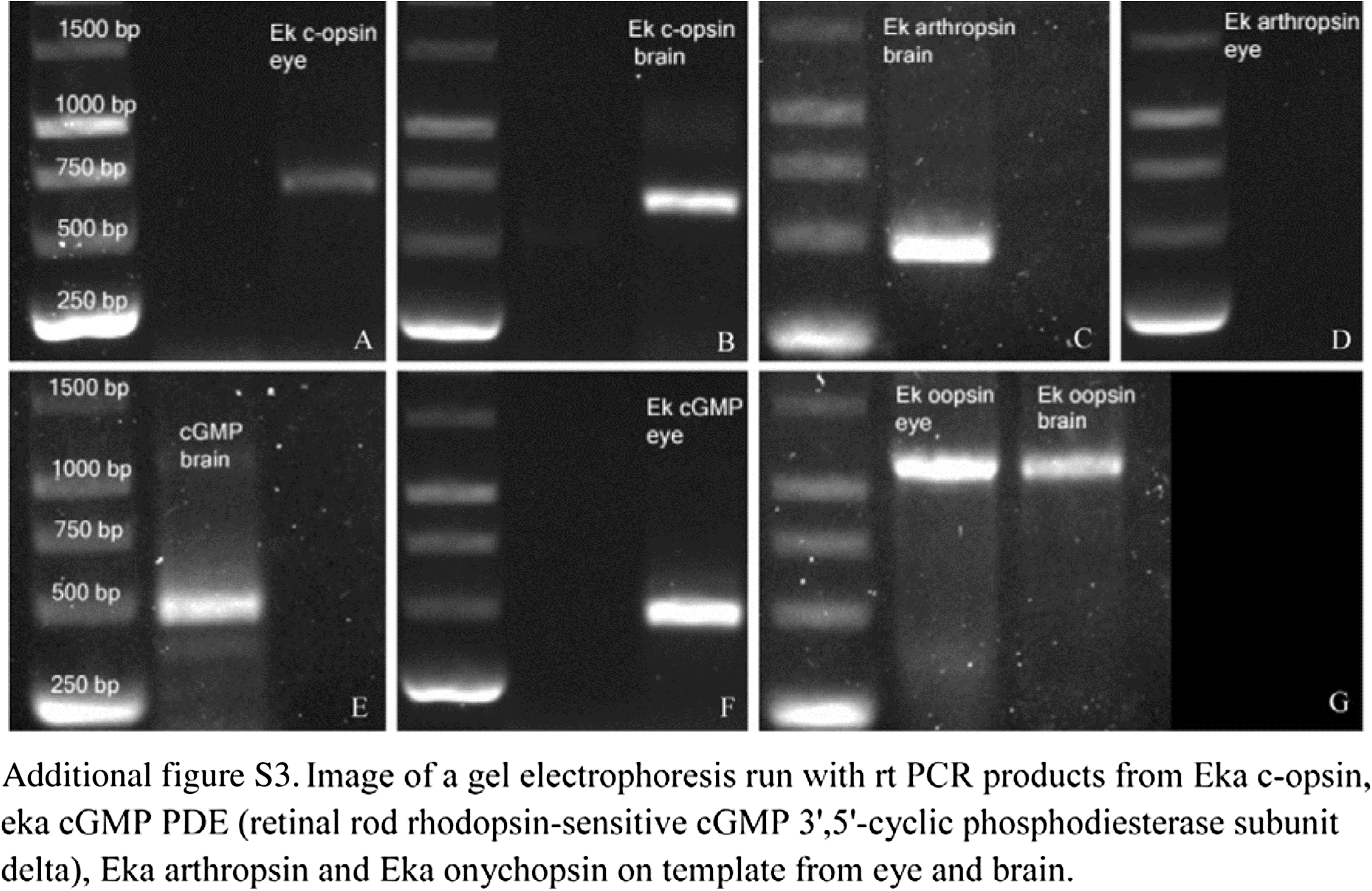

Supplement: Additional file 3: Figure S3 — Image of a gel electrophoresis run with rt PCR products from Eka c-opsin, eka cGMP PDE (retinal rod rhodopsin-sensitive cGMP 3’,5’-cyclic phosphodiesterase subunit delta), Eka arthropsin and Eka onychopsin an template from eye and brain. [file 1471-2148-13-186-S3.tiff]
